# Barriers to Person-Centered Chronic Kidney Disease Care: A Qualitative Study of Multidisciplinary Nephrology Clinicians

**DOI:** 10.1159/ajn/ablag007

**Published:** 2026-09-01

**Authors:** In-Gu Kang, JoAnn S. Oliver, Nayoung Kim, Ashley Abawi, Audrey Boahemaa Kusi, Felecia G. Wood, Pamela P. Foster, Sharlene D. Newman

**Affiliations:** a Alabama Life Research Institute, The University of Alabama, Tuscaloosa, AL, USA; b Capstone College of Nursing, The University of Alabama, Tuscaloosa, AL, USA; c Department of Health Science, The University of Alabama, Tuscaloosa, AL, USA; d Department of Community Medicine and Population Health, The University of Alabama, Tuscaloosa, AL, USA

**Keywords:** Chronic kidney disease, Dialysis decision-making, Patient education, Barriers to chronic kidney disease management, Qualitative research

## Abstract

**Introduction:**

The clinician-identified barriers to person-centered chronic kidney disease (CKD) care remain poorly characterized among socioeconomically disadvantaged populations. We examined nephrology clinicians’ perspectives on patient preparation, dialysis decision-making, and structural barriers to inform multilevel intervention.

**Methods:**

A qualitative descriptive study using semi-structured interviews and thematic analysis included 22 English-speaking nephrology clinicians recruited via snowball sampling. Transcripts were analyzed thematically.

**Results:**

Three themes emerged: first, CKD awareness, education, and preparedness – patient awareness was uneven and often absent even under nephrology care; education was multidisciplinary and stage sensitive but emotionally constrained; second, dialysis decision-making – planned outpatient initiation was distinguished from unplanned emergency starts, the latter associated with reduced patient choice and worse outcomes; modality discussions typically began at an estimated glomerular filtration rate of approximately 20 mL/min/1.73 m^2^ but timing varied widely; nephrology clinicians sometimes omitted home dialysis options when patient support seemed uncertain; and family involvement was central to adherence and decision quality; and third, barriers, supports, and clinical management – nonadherence reflected structural barriers, financial hardship, health literacy, transportation, and delayed referral, rather than motivation, while the clinician-patient relationship was a modifiable adherence determinant limited by understaffed support services.

**Discussion:**

Nephrology clinicians identified patient awareness, education timing, provider variability, and structural barriers as modifiable determinants of inequitable CKD outcomes. Multilevel intervention targeting pre-dialysis education, primary care coordination, reimbursement reform, and social support infrastructure is needed.

## Introduction

The period between chronic kidney disease (CKD) diagnosis and kidney failure is critical, requiring patients to navigate complex symptoms, comorbidities, and high-stakes treatment decisions [1]. Consistent with current Kidney Disease: Improving Global Outcomes (KDIGO) nomenclature recommendations, we use “kidney failure” and “kidney replacement therapy” rather than “end-stage renal disease” and “renal replacement therapy” throughout this manuscript [2]. CKD affects approximately 35.5 million US adults yet remains profoundly underrecognized: national estimates indicate approximately 90% of affected adults are unaware of their diagnosis overall [3], with awareness even lower in early-stage disease, only 3.2% among patients with stage 1–2 CKD, rising to only 10.5% among patients with stage 3–4 (moderate-to-severe) CKD [4, 5].

Person-centered care, grounded in patient preferences, shared decision-making, and life goals, is the recommended standard for supporting therapeutic engagement, symptom management, and adherence [6–8]. This distinction matters conceptually: while patient-centered care emphasizes clinical engagement and functional outcomes, person-centered care extends this focus toward what is meaningful to the individual, encompassing autonomy, dignity, family and social context, and life participation beyond biomedical markers [9]. Global survey data indicate that patients with CKD and their families prioritize symptom management and quality of life, whereas clinicians tend to prioritize clinical outcomes such as mortality and hospitalization [9], underscoring the need for nephrology care models that explicitly incorporate the person-centered perspective.

Within kidney care specifically, shared decision-making has been shown to remain inconsistently implemented across the advanced CKD and kidney failure trajectory, with variation in when, how, and by whom these conversations are initiated [10], and decision aids designed specifically for kidney failure treatment selection have been identified as an underused but effective tool for supporting patients and clinicians through this process [11]. However, for many patients, these conditions are absent well before dialysis initiation.

The transition to kidney replacement therapy is among the most consequential decisions in chronic disease management, with lasting implications for quality of life, functional independence, and survival. Clinical guidelines call for patients to be active, informed partners in modality selection [6], requiring adequate time and emotional readiness to deliberate. When education is delayed or fragmented, this foundation collapses. The unplanned or “crash” dialysis start, emergency initiation without prior access creation or modality planning, is a clear indicator of failure, associated with higher in-hospital mortality, greater central venous catheter use, and reduced access to home-based therapies [12–14].

Multiple barriers undermine person-centered care. Limited health literacy, affecting almost 25% of individuals with CKD, contributes to high mortality, reduced transplant access, missed sessions, and elevated costs [15, 16], compounded by poor cognition, cultural differences, and socioeconomic disparities [17–19]. System-level barriers include fragmented care, absent standardized pre-dialysis education, and inadequate multidisciplinary support [20–22]. Provider-level constraints, time pressure, resource shortages, and variability in modality counseling, add complexity only partially addressed in existing research [17–19].

Although patient and primary care perspectives on CKD management have been examined [23, 24], nephrology clinicians’ views on person-centered care for socioeconomically disadvantaged patient populations remain underexplored. This gap is particularly significant in Alabama, a state marked by high poverty, low educational attainment, and pronounced racial disparities in CKD burden [25]. Notably, much of this care is delivered through academic medical centers and large multi-provider group practices with substantial clinical infrastructure; the disadvantage patients face in this context is therefore primarily one of patient-level socioeconomic deprivation within a well-resourced health system, rather than system-level scarcity of clinical resources.

This qualitative study examines perspectives of 22 nephrology clinicians across diverse urban and rural settings, characterizing clinician-identified practices, barriers, and facilitators across three domains: patient awareness and preparation; dialysis decision-making; and structural and relational conditions shaping care. The study aimed to identify modifiable targets at the intersection of clinical education, system design, and health equity.

## Methods

Study reporting followed the Consolidated Criteria for Reporting Qualitative Health Research (COREQ) (online suppl. material S1; for all online suppl. material, see https://doi.org/10.1159/ajn/ablag007) [26]. This study used a qualitative descriptive design [27, 28], employing thematic analysis following Braun and Clarke’s [29] method, to explore nephrology clinicians’ perspectives on person-centered CKD care. Qualitative description was selected because it is well suited to generating a comprehensive, low-inference summary of clinician-identified barriers and facilitators in participants’ own terms, directly informing multilevel intervention design, without requiring the deeper theoretical or latent-level interpretation associated with more interpretivist qualitative traditions.

This study was conducted as formative research intended to inform the subsequent development of an intervention aimed at reducing racial disparities in CKD care and increasing utilization of home-based dialysis modalities. This downstream purpose informed the construction of the interview guide, which included specific content on home dialysis decision-making and modality selection (online suppl. material S2), and readers should consider that this orientation may have shaped both the questions asked of participants and the analytic emphasis placed on barriers to home dialysis uptake during interpretation.

### Participant Selection

Nephrology clinicians were recruited using snowball sampling. Initial participants were identified through professional networks at the University of Alabama at Birmingham (UAB) and the broader Alabama nephrology community and asked to refer colleagues. The resulting sample spanned multiple practice settings and regions within Alabama; however, because participants were recruited through referral within an interconnected professional network, this reflects the composition of that network rather than a deliberate sampling strategy for diversity, a limitation we address below. Of 96 nephrology clinicians invited by email and telephone, 22 nephrology clinicians participated. Of the 74 who did not participate, 8 explicitly declined, citing scheduling conflicts and time constraints, and the remaining 66 did not respond to the invitation.

### Data Collection

Interview questions were developed based on relevant literature spanning the major content areas addressed across the guide’s three domains and through iterative discussion among the research team. Questions in domain 1 (dialysis decision-making process) and domain 2 (patient-level reflective questions) addressing shared decision-making and modality selection were informed by evidence on patient and clinician preferences for shared and informed decision-making in kidney replacement therapy [30] and on patients’ psychosocial readiness for, and satisfaction with, modality selection [31]. Questions in domain 2 and domain 3 addressing patient preparedness, adherence, and information provision were grounded in established health literacy frameworks and evidence linking low health literacy to poorer CKD-related outcomes [32, 33]. Questions in domain 3 (practice context, care coordination, and nephrologist background) addressing referral timing and care coordination were informed by literature on individualized, disease-stage-based approaches to CKD management and documented disparities in CKD care trends [34, 35]. The interview guide was pilot tested with two nephrologists prior to the commencement of formal data collection and refined accordingly based on their feedback. The final semi-structured interview guide, comprising these three domains, is provided in the supplementary document (online suppl. material S2).

Authors J.S.O., F.W., and P.P.F. conducted individual semi-structured interviews from March 2023 to April 2024. Interviews were conducted by telephone. Only the participant and interviewer were present during interviews. Each interview lasted approximately 1 h. Participants received a USD 25 gift card in recognition of their time. All interviews were digitally audio-recorded with participant permission, transcribed verbatim by research team members, and checked for accuracy. Field notes were taken during interviews. Consistent with the aims of qualitative descriptive research, data collection continued until thematic saturation, the point at which no new concepts relevant to the study’s descriptive aims emerged from additional interviews was achieved. After approximately 20 interviews, recurring patterns were consistently identified across participants’ accounts and no new concepts emerged; 2 additional interviews were conducted to confirm saturation, yielding a total of 22 participants.

### Coding and Data Analysis

Transcripts were imported into the NVivo software (Lumivero, version 15) [36]. Consistent with thematic analysis following Braun and Clarke’s [29] six-phase method, two researchers (I.-G.K. and A.A.) independently conducted line-by-line coding of each transcript after familiarizing themselves with the data. The two researchers then met to compare codes, discuss and reconcile discrepancies, and reach consensus on the coding framework, an approach consistent with establishing coding consistency in qualitative descriptive research. Codes were grouped into preliminary themes and subthemes. Two additional research team members (N.K. and A.K.) reviewed the developing thematic structure and provided critical feedback; themes were iteratively refined and named accordingly through discussion among the coding team.

A thematic schema was developed to illustrate relationships among themes. Preliminary findings were presented to the full research team for consensus-based interpretation, in which team members reviewed the developing thematic structure, discussed alternative readings of the data, and reached agreement on the final coding and thematic framework, consistent with the consensus-oriented coding approach described above. This process was undertaken to ensure all relevant data patterns were captured and that interpretations were grounded in and consistent with the underlying data, rather than to independently verify findings across multiple data sources or analytic methods. As a form of respondent validation, a subset of participants (*n* = 5) reviewed preliminary findings and confirmed resonance with their experiences.

### Patient and Public Engagement

Patients and members of the public were not directly involved in the design of this study or the development of research questions, as the study recruited nephrology clinicians as participants rather than patients. CKD patients and patient advocacy representatives were not involved in recruitment or data collection. Findings will be disseminated through peer-reviewed publication, presentations at nephrology conferences, and direct communication of key findings to participating clinical sites. Insights from this study will inform the codesign of future patient-facing health literacy interventions, at which stage patients and community stakeholders will be engaged as active collaborators.

## Results

22 nephrology clinicians were interviewed, including 13 nephrology clinicians, 8 nurse practitioners, and 1 physician assistant. Clinical experience ranged from 1.5 to 45 years (mean: 15.4 years; median: 11.5 years); 12 (55%) were male. Most identified as White (*n* = 15), followed by Asian (*n* = 3), with African American, Hispanic/Latino, Indian, and Middle Eastern clinicians each represented by one participant (Table 1). Participants practiced across urban (primarily Birmingham and Huntsville), suburban, and rural settings, mostly in large group practices (6+ providers); 8 were affiliated with academic institutions. Practice types were mixed inpatient/outpatient (*n* = 14), outpatient only (*n* = 4), and inpatient only (*n* = 1); practice type was not reported for the remaining 3 participants (Table 1).

**Table 1.** Demographic characteristics of participants (*N* = 22)

Characteristic	*n* (%)
Practitioner type	
Nephrologist	13 (59)
Nurse practitioner/registered nurse	8 (36)
Physician assistant	1 (5)
Sex	
Female	10 (45)
Male	12 (55)
Race/ethnicity	
White	15 (68)
Asian	3 (14)
African American	1 (5)
Hispanic/Latino	1 (5)
Indian	1 (5)
Middle Eastern	1 (5)
Years of clinical experience	
<5	3 (14)
5–10	5 (23)
11–20	8 (36)
>20	6 (27)
M (SD)	15.4 (10.3)
Practice affiliation	
University/academic medical center	8 (36)
Kidney/nephrology group practice	14 (64)
Practice type	
Outpatient only	4 (18)
Inpatient only	1 (5)
Mixed inpatient/outpatient	14 (64)
Not reported	3 (14)
Practice location	
Urban only	4 (18)
Rural only	1 (5)
Mix of urban, suburban, and rural	17 (77)

Percentages may not sum to 100 due to rounding. M, mean; SD, standard deviation.

Thematic analysis following Braun and Clarke’s [29] method yielded three major themes: (1) CKD diagnosis awareness, patient education, and preparedness; (2) dialysis decision-making; and (3) barriers, supports, and clinical management of CKD. Themes and subthemes are described below; additional illustrative quotations appear in Table 2.

**Table 2.** Illustrative quotations for the three themes

**Theme 1. CKD diagnosis awareness, patient education, and preparedness**
Subtheme 1. CKD diagnosis awareness “There’s a slideshow to describe the different types of dialysis. There’s some hands-on stuff when they… They bring in models so that patients can see what’s going on, and some open discussion period as well.” (Male, ID6) “We interviewed people on dialysis, and I was amazed that a significant number of them did not realize that they had kidney disease, and they had been seeing a nephrologist. It didn’t click until they said, we got to start dialysis.” (Female, ID14) “These discussions are iterative. It’s like when a patient just shuts off, you can tell you’ve used the D word in the room and they just shut off. They actually don’t hear anything else for the rest of the appointment… Patient health literacy is different and the emotional engagement with one’s disease state is also different for different people.” (Male, ID13) “So I’m training internal medicine residents. So there, that I can propagate the knowledge because they have, so many of them have the same things. The medical residents say this, I’m like, no, that’s not going to work. So that’s one way at least that I can get this information out to people who then can propagate it to their patients. But other than that, I don’t have a good mechanism.” (Male, ID1)
Subtheme 2. Patient education “It’s a team effort. The dieticians are going to teach about diet. The social workers are going to educate about transportation options, transplant options. I educate a lot about medications, side effects. Okay. That’s great. What their other options are as far as home or peritoneal dialysis, the disease process. And then the nurses and the PCTs will do their own education, too.” (Female, ID4) “Well, early stage, three, three A, I don’t really talk about it because you don’t want to scare anybody. So at three A, stage two, stage one, your primary course of discussion is what steps are we taking to prevent the progression of your kidney disease? What are we doing? How are we managing your blood pressure? How are we managing your diabetes?” (Male, ID1) “But once you get below stage three B, your GFR drops below 30, you have to very aggressively start this. You have to mention it every time they come say, look, this is not right now, but this is coming down the road and you got to start talking, you need to start thinking about it.” (Male, ID1) “I would like to say that every patient I get, they make the decision to be in-center, because I really do try… Would you like to talk some more about home dialysis? Especially younger patients or somebody who has a spouse who’s really involved. Some of them had so many misunderstandings about home dialysis. One lady said, Uh-uh, I’d have to get rid of my dog… another told me, I’m not going to sit on a machine 12 hours… there’s a lot of misunderstandings, yes.” (Male, ID17)
Subtheme 3. Preparedness “I would sadly say that 50% of patients are prepared as best we can, which I would say is maybe not well-prepared, but pretty well-prepared. And another 50% of patients either because they either they suddenly discover unbeknownst to them for years that they had kidney failure, had no preparation. And some patients simply run and hide from the disease once they’re told that they have it, and so they will ignore the education and or not participate or engage for the future.” (Male, ID13) “Good question. Yeah, this is a very rough estimate for me, Doc, because like I said, when they come in they’re already in dialysis for months or years even. But I have a handful of patients that just recently started on dialysis. Let me say how prepared are they? Lets say maybe 60%. Okay, okay. Are most patients- I mean, they have the background, they know the diagnosis, nutrition, all the dialysis education part. But when they actually get connected to the machine and get the first treatment, they experience different side effects, different symptoms that they’re not that prepared for. It’s almost like a reality check, huh? Yeah, right. Yeah, because we won’t know how would they tolerate the dialysis, unless they get connected to the machine and start the dialysis.” (Female, ID10) “And similarly, as it’s time to start making some preparations for dialysis, while nobody embraces it or is excited about it, which by the way is a great challenge, understanding that no one would be excited about it, but asking them to prepare for something they don’t want to do. I will try to say that, ‘My job is to do two things for you at one time. Half of my job is to do everything I can to keep you off dialysis as long as possible. But the other part of my job is to do everything I can to make sure you’re ready for dialysis so that if you ever get there, you can do as well as possible.’” (Male, ID15)
**Theme 2. Dialysis decision-making**
Subtheme 1. Decision-making on dialysis initiation: clinic vs. hospital settings “But even in those patients over many years, eventually their kidney function will decline to the point where they require dialysis. And that decision was based on three or four factors, one of which is your laboratory values, which is to say you measure the creatinine, you measure the urine, and then you come up with what’s called a creatinine clearance or estimated GFR. And then, of course, you use clinical variables such as how is the patient feeling or their electrolyte problems… And then whether they have a functioning access in place and whether the access is ready, these are the different variables that would come into play with regards to the timing of initiation of dialysis.” (Male, ID1) “It’s a combination of factors. You first look at their labs. Typically, someone who starts dialysis, their GFR is less than 15, typically… Then after that, it’s based on whether they’ve developed symptoms that just can’t be controlled with medications. For example, if they’re developing way too much fluid buildup that we can’t use medications to get rid of, that would be one. If they’re starting to feel ill, nauseated, appetites dropping, they’re losing weight, that would be another indication.” (Male, ID21) “They meet our dieticians, when we get that on their first treatment we get a full set of labs… our dietician will sit down and talk to them about what their diet needs to look like… And we have a social worker too that helps us, are there any needs at home, do you need help getting to and from dialysis, and then of course our nurses and staff give them a rundown of what to expect.” (Female, ID18) “In the hospital, it’s like, ‘You got to sign this, or you don’t have to sign this, but hey, we need to sign this because we initiate dialysis.’ But as far as clinic basis, if somebody’s referred from our CKD clinics to our ESRD clinics, all that is handled the first day of dialysis, which can be a little overwhelming, but that’s generally how it’s handled.” (Male, ID17)
Subtheme 2. Multifactorial dialysis timing and shared modality decision-making through longitudinal education “So there’s multiple factors there. So obviously number one is their labs, so looking at their creatinine level, their BUN, the GFR that’s calculated, their electrolytes, potassium bicarbonate, things like that. Symptoms that the patient’s having as well, if they’re having nausea, vomiting, metallic taste in their mouth, poor appetite, really fatigue, sleeping all the time… And then obviously just what the patient wants out of their outcomes, if they are wanting to do dialysis or they wanting to go on hospice and understand that they may pass away.” (Male, ID2) “So I think it’s very important to understand and ask what your patients social and living situation is and who their support system is so that you can help also tailor your recommendation to their circumstances because your goal isn’t to get them on a specific modality. Your goal is that the modality works.” (Male, ID11) “Mostly the patient. If we don’t think that they’re capable of doing home dialysis, then we don’t usually talk much about it… If we think that they are capable or that someone in their family is willing to help them do it at home, we try to encourage usually peritoneal dialysis… we usually try to let them know that’s the way that most providers would choose if it was them in the seat.” (Female, ID20) “It should not be in the clinic, at least, this should not be the first time that this patient has heard about this. They should have been coming to clinic for years, and they will be coming to our office as necessary required. And every time they come, we should be talking to them a little bit about this, what’s happening, where are we moving? And so when the time actually comes, they should not be in a situation where they’re uneducated about it.” (Male, ID1)
Subtheme 3. Family role and patient-centered decision-making “I had a patient say, I feel great, and the daughter’s looking at me like, what is happening here? And she said, ‘he’s been sleeping 20 hours a day, he’s not eating.’ And all these things.” (Female, ID14) “If patients have a good support system, it’s much easier to get things accomplished, whether it’s we need to refer them somewhere and they need to get there, or even diet-related, making sure who’s cooking for them… And our patients that don’t have as much support, if any, it’s a lot harder to get things done.” (Female, ID18) “So, as you think about the most recent three to five patients that you’ve dealt with, who actually made the decision regarding the type of dialysis - the patient, the physician, the family? Almost 90% of the time it’s the patient.” (Male, ID7) “We have a lot of older women who… say, I’ll never do dialysis. I’ll never do dialysis. And we’ll let them say that up to a point… I am not trying to scare you, but there will come a point where we say, you either need to start dialysis or I need to call hospice. I think sometimes they don’t realize that it’s one or the other. It’s either life or death, and death is fine. You are perfectly able to choose that route.” (Female, ID14)
**Theme 3. Barriers, supports, and clinical management of CKD**
Subtheme 1. Disease complexity, adherence, and treatment burden “The medical challenges are that most people, not all, most people with CKD have multiple medical problems. They often have diabetes, high blood pressure. They often have heart disease. And so that’s the greatest complexity. Another is many, but not all, are older in age, and so they have age-related medical problems and functional limitations. I think social determinants of healthcare play a role. I think that diets that people have had their entire life, some medications are cost-prohibitive.” (Male, ID15) “But definitely see a lot of issues with adherence from all of it. And whether it be in the CKD population, them not taking medications appropriately, them not following low salt diet and not realizing that it’s way easier now than what it will be if you don’t do these things.” (Male, ID2) “Compliance is less than 25% of the patients are compliant. And some of it is just out of education, poverty. It’s just multiple issues. They’re not compliant at all.” (Male, ID7) “…the comorbid conditions, like that terrible diabetes or terrible blood pressure, or really bad bone disease or cardio heart disease, lung disease, their teeth. Dental issue is a big problem. The dental care needs to be taken care of, otherwise, they cannot get a transplant.” (Male, ID7)
Subtheme 2. Psychological impact, fear, and social experience of dialysis “Because when they’re scared, they’re likely to listen. And it is imperative, imperative that you get somebody at that visit besides them, usually a spouse, but somebody who can help the shock of all this. And you know how it’s you get a diagnosis of cancer, you’re not listening to the next things that are said.” (Male, ID9) “Yes. Sometimes we do get patients that are in denial. That happens more when we have an acute kidney injury patient, an AKI patient. That happens a lot more frequently in that population than it does those who have had some time to prepare for this mentally.” (Female, ID4) “The other thing that we recognized was that the in center dialysis was actually a social experience. It was pretty much the only social experience that these people had, because either they were old and they weren’t working or they had lost their employment. This became a real network for them to communicate.” (Male, ID2) “Yeah, they can’t comprehend that one fine day they were fine doing whatever they had to do, living their life, and that the next day, I’m telling them that they’re going to have to start dialysis and that they were remaining on dialysis the rest of their life.” (Male, ID1)
Subtheme 3. Structured, systemic, and logistic barriers to care “I think, probably, the biggest issue is the logistics of it. If it’s peritoneal dialysis, how are they going to get the training, and who’s going to help them at home? In-center hemodialysis, how are they going to get there three times a week? Logistics of getting those things done, I think, is the biggest… Some of the biggest barriers that we deal with is helping them figure out how to fit this within their lifestyle.” (Male, ID6) “Yes. That is probably my biggest. When I have a medication that I know works for the patient. I know it worked for them, and then the insurance just ‘We’re not going to cover that medication anymore. You’ve got to put them on this medication.’” (Male, ID17) “Would say one of the biggest challenges is just the demands of the healthcare system on the patient. Most of our patients with CKD have multiple comorbidities, so they’re not just seeing a kidney doctor, they’ll be seeing at least two other doctors, a primary care doctor and often another subspecialist, and they’re already fairly sick from that perspective.” (Male, ID5) “I think that we would do well if we were not so greedy, and whether that’s dialysis clinics… DaVita is greedy as all hell… I think healthcare should be a right, not a privilege.” (Male, ID9)
Subtheme 4. Relational supports, family involvement, and clinical foundations of care “So it’s trust that has the most to do with adherence, because if adherence is problematic because of finances, if a patient trusts you, they can tell you, ‘I just can’t afford it, doc, I need something cheaper.’” (Male, ID9) “Well, I mean the family plays a huge role because a lot of times family members can’t help if they want to do home therapy and it’s easier for them just to drop them off at the clinic so they don’t have to deal with that responsibility and the supplies.” (Female, ID8) “If patients have a good support system, it’s much easier to get things accomplished, whether it’s we need to refer them somewhere and they need to get there, or even diet-related, making sure who’s cooking for them, are they cooking for themselves, do they have access to being able to grocery shop.” (Female, ID18) “The social worker and dietician helped tremendously to try and navigate that. And a lot of the different companies have good assistance programs now.” (Male, ID2)

### Theme 1: CKD Diagnosis Awareness, Patient Education, and Preparedness

#### Subtheme 1: CKD Diagnosis Awareness

Patient awareness of CKD ranged from detailed comprehension to shock, denial, and minimal disease recognition. CKD’s lower public profile compared with cancer or heart disease left many patients unaware of deteriorating kidney function and vulnerable to misconceptions, a pattern recurring across practice settings. Knowledge gaps were compounded by inaccessible materials, language barriers, and frequent exclusion of family members. Patients sometimes remained unaware even under specialized kidney care: *“We interviewed people on dialysis, and I was amazed that a significant number of them did not realize that they had kidney disease, and they had been seeing a nephrologist. It didn’t click until they said, we got to start dialysis”* (Nurse Practitioner, ID14). Participants advocated multimodal teaching including visual aids, hands-on models, and plain language materials but recognized these alone were insufficient when emotional barriers intervened: *“These discussions are iterative. It’s like when a patient just shuts off, you can tell you’ve used the D word in the room and they just shut off”* (Nephrologist, ID13).

#### Subtheme 2: Patient Education

Education was a deliberate, longitudinal process distributed across a multidisciplinary team: *“It’s a team effort. The dieticians are going to teach about diet. The social workers are going to educate about transportation options, transplant options. I educate a lot about medications, side effects… What their other options are as far as home or peritoneal dialysis”* (Nurse Practitioner, ID4). Delivery was stage sensitive. General awareness conversations began early, with dialysis-specific preparation intensifying at a GFR below 30 mL/min/1.73 m^2^ and repeated at each visit, since patients rarely absorbed major information at once. Despite these approaches, participants described fragmented care, crash presentations, and patient fear as ongoing constraints on educational effectiveness.

#### Subtheme 3: Preparedness

Preparedness depended more on patients’ emotional state and life circumstances than on the quality of educational materials. One participant’s 50% estimate captured a pattern of late diagnosis, fear-driven avoidance, and unplanned admission: *“I would sadly say that 50% of patients are prepared… And another 50%… either suddenly discover unbeknownst to them for years that they had kidney failure, had no preparation. And some patients simply run and hide from the disease”* (Nephrologist, ID13). The core challenge was sustaining engagement during asymptomatic periods: *“Half of my job is to do everything I can to keep you off dialysis as long as possible. But the other part of my job is to do everything I can to make sure you’re ready for dialysis so that if you ever get there, you can do as well as possible”* (Nephrologist, ID15).

### Theme 2: Dialysis Decision-Making

#### Subtheme 1: Decision-Making on Dialysis Initiation – Clinic versus Hospital Settings

Participants distinguished planned outpatient dialysis starts from unplanned emergency initiations, noting fundamentally different experiences and outcomes. Planned starts allowed gradual, multi-visit assessment of kidney function, symptoms, and patient preferences, giving patients time and agency. Emergency starts compressed all decisions into a single urgent encounter, often with no prior education or access in place: *“In the hospital, it’s like, ‘You got to sign this… because we initiate dialysis.’ But as far as clinic basis, if somebody’s referred from our CKD clinics to our ESRD clinics, all that is handled the first day of dialysis”* (Nephrologist, ID17). Unplanned starts were associated with lower treatment acceptance, poorer adherence, and worse outcomes, as perceived by participants. Late referrals from primary care were consistently identified by participants as the primary perceived contributor to unplanned starts.

#### Subtheme 2: Multifactorial Dialysis Decision and Shared Modality Decision-Making

Timing of initiation and modality selection were identified as the two pivotal decisions in advanced CKD, both requiring early preparation. Initiation timing was individualized, incorporating declining estimated glomerular filtration rate (eGFR), electrolytes, symptoms, functional status, and patient preference, including comfort-focused care. Most participants initiated modality discussions at approximately eGFR 20 mL/min/1.73 m^2^, reasoning that early framing enabled genuine deliberation: *“It should not be in the clinic, at least, this should not be the first time that this patient has heard about this. They should have been coming to clinic for years… when the time actually comes, they should not be in a situation where they’re uneducated about it”* (Nephrologist, ID1).

Participants described modality choice as shaped by two coexisting, and at times competing, patterns. Several clinicians emphasized orienting the decision around the patient’s home situation, support, and personal priorities, framing their own role as ensuring the chosen modality would function well for that patient’s circumstances rather than steering the outcome: *“Your goal isn’t to get them on a specific modality. Your goal is that the modality works”* (Nephrologist, ID11).

Other accounts, however, revealed that clinicians made a preemptive judgment about feasibility that shaped which modalities were even raised with the patient: “*Mostly the patient. If we don’t think that they’re capable of doing home dialysis, then we don’t usually talk much about it… If we think that they are capable or that someone in their family is willing to help them do it at home, we try to encourage usually peritoneal dialysis… we usually try to let them know that’s the way that most providers would choose if it was them in the seat*” (Nurse Practitioner, ID20). Rather than a uniformly patient-driven process, modality selection therefore reflected an interplay between patient priorities and clinician judgments about feasibility that could constrain the options a patient was offered in the first place.

#### Subtheme 3: Family Role and Person-Centered Decision-Making

Person-centered care required supporting a sequence of decisions, including life-sustaining treatment versus the natural course of disease, transplantation versus dialysis, home versus in-center care, and peritoneal versus home hemodialysis. Early decisions constrained later options. Family members were key partners and often provided more accurate functional accounts than patients themselves: *“I had a patient say, I feel great, and the daughter’s looking at me like, what is happening here? And she said, ‘he’s been sleeping 20 hours a day, he’s not eating’”* (Nurse Practitioner, ID14). Involved family support predicted treatment engagement, though caregiver strain and skepticism could also generate tension. Dialysis refusal or withdrawal was universally accepted as a legitimate patient right. Participants further noted that current hospice eligibility rules require patients to discontinue dialysis in order to enroll in palliative hospice care.

### Theme 3: Barriers, Supports, and Clinical Management of CKD

#### Subtheme 1: Disease Complexity, Adherence, and Treatment Burden

CKD management was described as inherently demanding including polypharmacy, dietary restrictions, fluid limits, and dialysis schedules compounding one another. Participants described adherence as low, though their accounts reflected clinical impression rather than measured adherence data: one participant estimated, *“Compliance is [low] − less than 25% of the patients are compliant. And some of it is just out of education, poverty. It’s just multiple issues”* (Nephrologist, ID7). Participants attributed perceived nonadherence not to motivational deficits but to overlapping structural barriers, including financial hardship, limited health literacy, and competing caregiving responsibilities, conditions that would challenge adherence in any population. This burden was particularly pronounced among patients with diabetes, for whom dietary restrictions, fluid limits, and glycemic management demands compounded one another, creating adherence challenges that exceeded those faced by patients managing a single chronic condition.

#### Subtheme 2: Psychological Impact, Fear, and Social Experience of Dialysis

CKD diagnosis was described as delivering a shock comparable to cancer, leaving patients least able to absorb information precisely when it was most needed. Fear of machines, needles, and home management independence were ongoing barriers requiring proactive attention in education programs. A trusted support person at key appointments was identified as critical: *“Because when they’re scared, they’re likely to listen. And it is imperative… that you get somebody at that visit besides them… somebody who can help the shock of all this”* (Nephrologist, ID9). Participants also highlighted the dialysis center as a genuine social community, a dimension rarely addressed in clinical literature. Participants described patient deaths or hospitalizations as sources of real grief among peers, an emotional burden that clinical teams reported feeling ill-equipped to manage.

#### Subtheme 3: Structural, Systemic, and Logistical Barriers to Care

Structural, logistical, and financial obstacles were described as widespread and often the deciding factor in how treatment unfolded. Transportation was the most common barrier, especially for in-center hemodialysis requiring three weekly facility visits: *“I think, probably, the biggest issue is the logistics of it… In-center hemodialysis, how are they going to get there three times a week?… Some of the biggest barriers that we deal with is helping them figure out how to fit this within their lifestyle”* (Nephrologist, ID6). Insurance restrictions, medication costs, and language barriers compounded disengagement risk. Unplanned starts were linked to late referral and payment models that prioritize dialysis initiation over pre-dialysis education, which are system-level problems that individual clinicians can identify but not resolve.

#### Subtheme 4: Relational Supports, Family Involvement, and Clinical Foundations of Care

The long-term clinician-patient relationship was identified as the single most important determinant of adherence and decision quality, something participants actively cultivated rather than assumed: *“It’s trust that has the most to do with adherence, because if adherence is problematic because of finances, if a patient trusts you, they can tell you, ‘I just can’t afford it, doc, I need something cheaper’”* (Nephrologist, ID9). Trust functioned as a clinical tool, enabling discrimination between volitional and circumstantial nonadherence, each of which requires a different response. Family involvement extended support beyond the clinic but required active management given caregiver strain and occasional treatment skepticism. Social workers, health navigators, and financial assistance programs were essential, yet chronic understaffing concentrated their absence among the most vulnerable patients.

## Discussion

Interviews with 22 kidney disease clinicians revealed three major themes, demonstrating that care quality is shaped by relational, educational, structural, and equity-related conditions alongside clinical expertise. Findings highlight substantial variability in treatment option counseling, home modality recommendations, and primary care communication, each a modifiable intervention target. These themes are examined below across four analytic domains: CKD awareness; patient education and decision-making; nephrology-primary care integration; and barriers and supports. The first two domains address the two component parts of theme 1 (CKD diagnosis awareness, patient education, and preparedness) separately for analytic clarity; the third integrates theme 1’s education timing findings with theme 2 (dialysis decision-making) to develop our central argument that these reflect a single systems-level gap rather than separable problems; and the fourth corresponds to theme 3 (barriers, supports, and clinical management of CKD).

### CKD Awareness as a Foundational Intervention Target

CKD awareness is a foundational but underdeveloped component of disease management. Of approximately 35.5 million Americans with CKD, 90% are unaware of their diagnosis overall [3]; awareness is stage-dependent, with rates as low as 3.2% among patients with early-stage (stage 1–2) CKD and 10.5% among patients with moderate-to-severe (stage 3–4) CKD [4, 5]. This gap is both informational and motivational, shaping engagement with self-management, risk factor modification, and treatment decision-making [4]. Early awareness is particularly critical because CKD progression can be slowed, but not reversed, through lifestyle modification, comorbidity management, and pharmacologic intervention [37–40]. Delayed awareness therefore represents a missed clinical window with lasting consequences for disease trajectory and treatment options.

Awareness deficits extend to providers. Prior work has demonstrated persistent knowledge gaps among primary care providers (PCPs) [41, 42] and underutilization of evidence-based therapies known to slow CKD progression (e.g., renin-angiotensin-aldosterone system inhibitors in the form of angiotensin-converting enzyme inhibitors) and the angiotensin receptor blockers [43, 44], suggesting that delayed recognition and suboptimal early management are structurally embedded in the care continuum. Participants described variability in how and when CKD is communicated. These findings position CKD awareness as the first link in a chain running from early detection through education to informed modality choice, developed further below.

### Patient Education and Decision-Making

Awareness is necessary but insufficient without timely, comprehensive education supporting meaningful care engagement. Variability in education content and timing contributed directly to differences in preparedness, with some patients entering modality discussions with limited disease understanding, an inconsistency constraining shared decision-making and reinforcing default selection of in-center dialysis when alternatives were inadequately introduced.

Evidence links comprehensive patient education to improved clinical and decision-related outcomes [45], including delayed disease progression [46], better dialysis preparation [47], and more effective vascular access use [48]. The therapeutic landscape underlying this progression-delaying potential has itself evolved substantially in recent years: sodium-glucose cotransporter-2 inhibitors are now recommended as part of first-line pharmacotherapy for slowing CKD progression across a broad range of patients, with or without diabetes [49]. Patient understanding of, and adherence to, these newer disease-modifying therapies represents an increasingly important, though currently unaddressed, dimension of the education gaps our participants described. Such education enables shared decision-making by aligning treatment choices with patient values and life circumstances [45]. Clinicians described the necessity of foundational CKD understanding before modality discussions, yet delivery timing varied widely across participants and practices, particularly for patients first learning of their diagnosis during hospitalization. This variability, not the content of education itself, was the primary constraint participants described; we examine its structural origin below.

Structured preparation must begin well in advance of dialysis need; late delivery reflects a systemic failure to afford patients adequate deliberation time. That fragmented care, crash presentations, and patient fear persisted, although deliberate, multidisciplinary educational efforts (see Results) suggest these constraints reflect upstream systemic conditions rather than shortcomings in individual clinician-patient encounters; refining encounter-level communication strategies alone is therefore unlikely to resolve them. Persistent knowledge and access gaps at patient and provider levels [50, 51] reinforce that education cannot be addressed in isolation from system-level constraints. Comprehensive, early, and standardized patient education is a central intervention target for improving CKD management, shared decision-making, and equity in treatment outcomes.

### Nephrology and Primary Care Integration

Provider-level variability in modality education cannot be understood apart from the inherently shared structure of CKD care between PCPs and nephrologists. PCPs anchor early identification and management; nephrologists manage advanced disease and prepare patients for kidney replacement therapy. However, this transition is frequently delayed or poorly coordinated [52–54], and participants’ accounts (see Results) suggest that late referral, and the unplanned dialysis starts it produces, is not a problem solvable at the point of care alone: it originates in the structural handoff between primary and specialty care and therefore requires system-level rather than individual-encounter-level remediation, with a substantial proportion of patients entering nephrology care late in the disease course or initiating dialysis without prior nephrology engagement [55].

These patterns reflect not only breakdowns in communication, but also structural constraints, including a growing nephrology workforce shortage [56]. Primary care is not simply a referral source but a critical, ongoing site of CKD management. Participants located the source of the timing variability described above squarely in this transition: many reported initiating dialysis discussions at an eGFR of approximately 20 mL/min/1.73 m^2^, while others waited until initiation was imminent, and delayed referral was consistently identified as the primary perceived upstream contributor to crash dialysis starts, which some participants estimated affected a majority of patients in some practices.

A related, well-intentioned gatekeeping pattern compounded this constraint: at least one participant, a nurse practitioner, described a practice of not raising home dialysis as an option when the patient or family’s capability to manage it at home seemed uncertain (Table 2, ID20), further narrowing an already compressed decision window before the patient had an opportunity to weigh in. That this account comes from a nurse practitioner rather than a nephrologist is itself notable: it suggests this gatekeeping practice may not be confined to physicians exercising formal authority over modality prescribing, but may instead reflect a broader clinical norm shared across the care team, one in which any team member, regardless of formal decision-making authority, may preemptively narrow the options presented to a patient based on their own judgment of feasibility. If gatekeeping operates at the level of team culture rather than individual physician discretion, interventions aimed at expanding home dialysis uptake will need to address decision-making norms across the full care team, not nephrologists alone.

Given the analytic weight of this single account, however, we treat this as a hypothesis-generating observation warranting confirmation in future work with a larger sample of nonphysician participants, rather than a confirmed pattern across our data. This pattern is consistent with kidney-specific scoping review evidence showing that interventions supporting shared decision-making for treatment modality decisions in advanced kidney disease remain inconsistently implemented and evaluated, with only about one-third of identified interventions reported as adopted into clinical practice [10].

Taken together, these findings describe a single systems-level failure rather than three independent problems: low CKD awareness, variable education timing, and delayed nephrology referral are sequential symptoms of the same structural gap, fragmented, poorly incentivized coordination between primary and specialty care, rather than separable deficits in individual clinician communication or patient understanding. Addressing awareness or education in isolation is therefore unlikely to succeed without standardizing referral timing and modality education content across the primary-to-specialty transition. Strengthening collaboration through structured referral pathways, shared care models, and coordinated pre-dialysis education, beginning in primary care and continuing through nephrology, is accordingly a high-priority, systems-level intervention target, not a set of discrete communication fixes. Structured decision aids designed specifically for kidney failure treatment selection offer one concrete, evidence-based mechanism for standardizing this process at the point of modality decision-making, since kidney services are known to vary considerably in how they involve patients in these decisions [11]. Strengthening collaboration through structured referral pathways, shared care models, and coordinated pre-dialysis education is essential, not optional, to ensure timely, guideline-coordinated care across the CKD trajectory.

### Barriers, Supports, and Implications for Intervention Design

Adherence failures concentrated in patients with diabetes managing simultaneous dietary, fluid, and glycemic management demands (see Results) confirm that nonadherence is a multifactorial structural-clinical phenomenon; structural remediation, not behavioral or educational programming alone, is required to meaningfully improve adherence in this population. Transportation demands, insurance restrictions, medication costs, language barriers, under-resourced transplant programs, and hospice eligibility rules that require patients to discontinue dialysis in order to enroll in palliative care (see Results) each represent discrete structural failures requiring policy-level remediation; the hospice eligibility barrier in particular reflects a policy-level conflict between two guideline-endorsed care pathways, dialysis, and palliative/hospice care, rather than a deficiency in individual clinician communication, and is unlikely to be resolved without reform at the regulatory or reimbursement level. The emergence of in-center dialysis as a primary social community carries direct implications for home dialysis promotion. Patients embedded in dialysis unit social networks face social as well as logistical barriers to modality transitions, necessitating peer mentorship and psychosocial support alongside practical preparation. Chronic understaffing of navigators, social workers, and financial assistance programs constrained the patients who needed them most, positioning sustained investment in these roles as among the highest-yield system-level targets. Kidney replacement therapy remains the single most consequential clinical decision once kidney failure is established, underscoring that the entire infrastructure of CKD care exists to support choices whose stakes are irreducibly high.

## Limitations

This study has several limitations. Recruitment was limited to English-speaking participants, potentially underrepresenting linguistically diverse settings. Additionally, recruitment originated within UAB and the greater Alabama nephrology network, with expansion through snowball referrals. Snowball sampling characteristically draws participants from within an interconnected professional network, clinicians who trained together refer patients to one another or share institutional affiliation and culture, and therefore tends to produce a more homogeneous sample rather than one that is independently diverse. Although our final sample spanned multiple practice settings, regions, and both academic and community-based practices, this diversity should be interpreted as a feature of the particular network sampled rather than evidence that the method itself yielded a broadly representative or unbiased cross section of nephrology clinicians. Perspectives from clinicians outside this network, including those trained at other institutions, practicing in other states, or without professional ties to the seed participants, may differ in ways this study cannot capture. Generalizability beyond the sampled clinical contexts should therefore be considered limited.

Our sample also combined nephrologists (*n* = 13), nurse practitioners (*n* = 8), and one physician assistant, rather than nephrologists alone. While this multidisciplinary composition allowed us to capture perspectives across the nephrology care team, findings attributed to “nephrology clinicians” collectively may obscure role-specific differences in perspective, decision-making authority, or practice; our sample size did not permit systematic comparison across roles, and the gatekeeping finding attributed to a single nurse practitioner participant (see Discussion) illustrates the risk of drawing role-specific conclusions from a sample of this composition and size.

Relatedly, our characterization of the study population as socioeconomically disadvantaged or “resource-poor” refers specifically to patient-level deprivation, poverty, low educational attainment, and transportation and financial barriers, within a health system that itself includes substantial clinical infrastructure (an academic medical center and large multi-provider group practices). Findings may not generalize to settings in which “resource-poor” instead describes system-level scarcity, such as rural single-provider clinics, safety-net systems with constrained clinical infrastructure, or nephrology care in low- and middle-income countries, where clinicians may face different structural constraints than those described here.

Of 96 nephrology clinicians invited, 22 participated, fewer than our original recruitment target of approximately 30. While saturation was achieved within this sample (see Methods), a larger sample may have surfaced additional or more varied perspectives, particularly from clinicians in practice settings or role types underrepresented among our 22 participants. We are also unable to fully characterize potential nonresponse bias: of the 74 clinicians who did not participate, 8 explicitly declined, citing scheduling conflicts and time constraints, while the remaining 66 did not respond to the invitation at all. We do not have demographic, practice-setting, or role information for nonparticipants and therefore cannot assess whether they differed systematically from participants in ways that might have shaped which perspectives are represented in our findings.

This study was designed as formative groundwork for a planned intervention aimed at increasing home-based dialysis utilization, and the interview guide was constructed accordingly, with specific attention to modality decision-making and barriers to home dialysis. This orientation may have shaped which topics were probed most closely and which patterns were emphasized during analysis; findings regarding barriers to home dialysis, including the gatekeeping pattern discussed above, should be interpreted with this prespecified analytic lens in mind, rather than as emerging from a neutral, nondirectional inquiry into CKD care generally.

As a qualitative study, findings reflect participants’ perceptions rather than objectively measured outcomes. Sex- and gender-based analyses of clinician perspectives were not conducted; future research should examine whether gender shapes communication practices or modality counseling patterns. Future research should incorporate patient and caregiver perspectives, employ role-stratified or multi-network sampling to address the limitations described above, examine the effectiveness of specific health literacy interventions on home dialysis uptake and racial disparity reduction, and evaluate whether structural reforms in referral timing and reimbursement produce the outcome improvements our findings suggest are achievable.

## Acknowledgments

The authors gratefully thank all participating nephrology clinicians for their time and willingness to share their perspectives. We also thank Sabrena Andrews for her assistance with participant recruitment. Finally, we thank the reviewers for their thorough, constructive feedback, which substantially strengthened this manuscript.

## Statement of Ethics

This study was approved by the University of Alabama Ethics Committee, Approval No. 21-05-4671. All study procedures were conducted in accordance with the ethical principles of the World Medical Association Declaration of Helsinki. Verbal informed consent was obtained from all nephrology clinician participants prior to their participation in this study. Consent was audio-recorded at the start of each interview, and a waiver of written documentation of consent was approved by the University of Alabama Ethics Committee.

## Conflict of Interest Statement

The authors have no conflicts of interest to declare.

## Funding Sources

The study was supported by a grant from CVS Kidney Care, LLC. The funders had no role in study design, data collection, analysis, interpretation, or the writing of this manuscript. This study was conceived as formative research supporting a planned intervention, funded by the same source, and aimed at increasing home-based dialysis utilization; while the funder did not direct the design, conduct, or interpret the specific study results, readers should be aware of this programmatic relationship when considering the study’s framing and emphases.

## Author Contributions

Research idea and study design and supervision or mentorship: S.D.N., F.G.W., J.S.O., and P.P.F.; data acquisition: J.S.O., F.G.W., and P.P.F.; data analysis/interpretation: I.-G.K., A.A., N.K., A.B.K., F.G.W., and J.S.O. Each author contributed important intellectual content during manuscript drafting or revision and agreed to be personally accountable for the individual’s own contributions and to ensure that questions pertaining to the accuracy or integrity of any portion of the work, even one in which the author was not directly involved, are appropriately investigated and resolved, including with documentation in the literature if appropriate.

## Data Availability

The data supporting the findings of this study consist of audio recordings and de-identified transcripts from semi-structured interviews with nephrology clinicians. These data are not publicly available due to the sensitive nature of the information shared by participants and to protect participant confidentiality, as participants were not consented for unrestricted data sharing. De-identified data may be made available to qualified researchers upon reasonable request to the corresponding author, subject to institutional review board approval and execution of a data use agreement.
